# Fusion of Enhanced and Synthetic Vision System Images for Runway and Horizon Detection

**DOI:** 10.3390/s19173802

**Published:** 2019-09-03

**Authors:** Ahmed F. Fadhil, Raghuveer Kanneganti, Lalit Gupta, Henry Eberle, Ravi Vaidyanathan

**Affiliations:** 1 Department of Electrical Engineering, University of Kirkuk, Kirkuk, 36001, Iraq; 2 Department of Electrical and Computer Engineering, Southern Illinois University, Carbondale, IL 62901, USA; 3 Department of Biomedical Engineering, University of Reading, Whiteknights, Reading RG6 6AH, UK; 4 Department of Mechanical Engineering, Imperial College London, London SW7 1AL, UK

**Keywords:** unmanned aircraft (UAV), sensing, intelligent transportation, image fusion, signal alignment, runway detection, image registration, wavelet transform, Hough transform

## Abstract

Networked operation of unmanned air vehicles (UAVs) demands fusion of information from disparate sources for accurate flight control. In this investigation, a novel sensor fusion architecture for detecting aircraft runway and horizons as well as enhancing the awareness of surrounding terrain is introduced based on fusion of enhanced vision system (EVS) and synthetic vision system (SVS) images. EVS and SVS image fusion has yet to be implemented in real-world situations due to signal misalignment. We address this through a registration step to align EVS and SVS images. Four fusion rules combining discrete wavelet transform (DWT) sub-bands are formulated, implemented, and evaluated. The resulting procedure is tested on real EVS-SVS image pairs and pairs containing simulated turbulence. Evaluations reveal that runways and horizons can be detected accurately even in poor visibility. Furthermore, it is demonstrated that different aspects of EVS and SVS images can be emphasized by using different DWT fusion rules. The procedure is autonomous throughout landing, irrespective of weather. The fusion architecture developed in this study holds promise for incorporation into manned heads-up displays (HUDs) and UAV remote displays to assist pilots landing aircraft in poor lighting and varying weather. The algorithm also provides a basis for rule selection in other signal fusion applications.

## 1. Introduction

The precise detection of runways is crucial for safely landing aircraft; nearly half of aircraft accidents are reported to occur during the final approach and landing stages [1]. While instrument landing systems have successfully been implemented to provide precise landing guidance, they are not available at all airports. Furthermore, smaller aircraft and fixed wing unmanned air vehicles (UAVs) often land in remote locations with only small runway strips available. Thus, there is a clear need to assist pilots and remote UAV operators using visual flight landing aids to detect runways accurately in varying weather conditions. Readily available imaging systems offer obvious potential to address this issue, but a single mode of image capture often does not fully convey all vital landing information in time critical situations. Fusion of ground sensor arrays (e.g., infrared cameras [2]) have been proposed to provide real-time input to UAVs, in particular in absence of GPS information [3]. Such systems can be enhanced through the fusion of image information from disparate heterogeneous sensors in real-time.

The convolution of output acquired from multiple sensors capturing complementary information has received significant attention in recent years. Techniques for fusing such information from images have been applied to a diverse range of fields where the volume of visual information would otherwise be difficult for humans to interpret. In medical imaging image fusion is usually performed at the pixel level, rather than using specific image features, as the emphasis is on increasing the visual contrast of different tissues that are more or less visible to particular imaging techniques such as X-ray, computed tomography, or magnetic resonance imaging. In [4] the non-subsampled contourlet transform (NSCT) was used in order to allow the researchers to apply two different fusion rules to the low- and high-frequency elements of medical images, while in [5] optimal coefficients are calculated for combining the images at multiple spatial resolutions, ensuring that features at different scales are treated consistently and that artifacts are reduced. Contrastingly, a persistent goal in remote sensing is to fuse a high spectral resolution multispectral (MS) image and a high spatial resolution panchromatic (PAN) image in order to produce an image with high resolution in both domains. In [6] this was achieved by using the curvelet transform to extract the directional details of the PAN image before injecting them into the upsampled MRA image, and [7] addresses the same problem via optimization, reconstructing a high-resolution MS image using a dictionary learned from the PAN image. Image fusion is also used in intelligent transport [8,9], where feature-based fusion, reliant on identifying stable characteristics that are present in multiple images, aids in the automated recognition of cars and other road vehicles. The previously described techniques are also employed in surveillance [10], low altitude remote sensing [11], and color visibility enhancement [12], among others. 

Very recent approaches such as that developed by Li et al. [13] have introduced weighted fusion strategies aimed at improving the robustness of detection against the size of object. Such approaches hold intriguing potential for the detection of weakly illuminated areas, which provide particular challenges in recognition which have not been fully addressed today [14]. Fusion of images with complementary vehicle control information has been also been introduced for UAVs [15], and algorithms combining infrared and visible spectrum images have been designed for target tracking [16].

The accurate detection of runways has received considerable attention in the literature, and a wide variety of techniques have been developed to detect runways in aerial images using the Hough transform and least squares to obtain boundary information [17], registering the runway with known reference images in different orientations [18], edge detection in conjunction with Hough transform line detection [19], and intensity and contrast of the runway and background [20]. Runways have also been detected in satellite images using texture properties [21], edge detection in conjunction with the Hough transform and chain codes [22], Helmholtz principle [23], shape and chroma features [24], as well as edge detection and fuzzy logic [25]. Detecting runways is also an important part of obstacle detection on runways [26,27,28]. Related efforts have also focused on horizon detection in low visibility conditions [26] and to identify the ground in images for emergency landings [29,30].

While image fusion represents an obvious approach for runway detection, Liu and Yu [31] argued that the majority of approaches today still rely on the assumption that source images are perfectly aligned. This makes them unsuitable for the application presented in this paper, where runway and horizon detection and enhancement must be performed using information from both enhanced vision system (EVS) and synthetic vision system (SVS) images of the runways. The EVS image is the runway as viewed with an infra-red sensor and the SVS image is a computer-generated view of the runway based on pre-existing map data and the plane’s GPS-determined location. These images require alignment in order to fully capture their complementary image information for aircraft runway detection and enhanced situational awareness.

**Figure 1:**
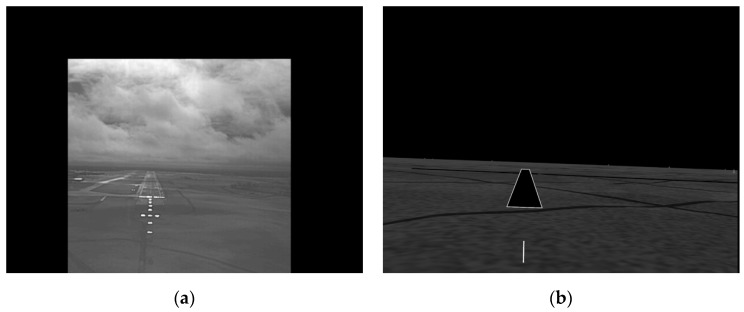
An example of (**a**) enhanced vision system (EVS) and (**b**) synthetic vision system (SVS) image frames.

Figure 1 shows examples of EVS and SVS images of a runway. The EVS image can normally be used by pilots during landing but its quality is badly reduced by adverse weather conditions, and it displays a smaller region of the horizon than the SVS. In contrast, the SVS image is not affected by the weather, but as it is not a ‘real’ image of the runway that shows features such as moving objects, the pilot cannot safely land the aircraft using it alone. The approach developed in this paper is to exploit the weather-invariant SVS image information to accurately detect the runway in the weather-dependent EVS image and thus generate an EVS-SVS composite image which contains information from both images. The EVS and SVS images are not originally aligned, and are therefore registered using runway and horizon features prior to fusion. Different fusion rules are developed to combine the EVS and SVS images and evaluated in the presence of varying levels of atmospheric turbulence.

The principal research objective is to create image frames that contain enhanced runway and surrounding information by fusing the EVS and SVS images so they can be incorporated into head-up displays (HUDs) or unmanned aerial vehicle (UAV) remote displays to assist pilots in safely landing aircraft. This objective requires both accurately detecting the runway in a sequence of frames through registration and the subsequent enhancement of the surrounding image region by fusing the EVS and SVS images. Information from the weather-independent SVS image is used to approximate the runway and horizon in the weather-dependent EVS image. The SVS image is aligned and fused with the EVS image. Since the most critical need for accurate runway detection is during the landing phase, the focus is on registering and fusing the EVS and SVS images when the aircraft is close to the runway. The procedure is made autonomous from frame-to-frame by deriving parameters from the SVS image. The performance of the fusion methodology is validated on a data set consisting of 1350 pairs of EVS and SVS frames of the runway acquired while an aircraft was landing, which was used in its entirety. Furthermore, additional subjective and objective evaluations are conducted using simulated EVS images with varying levels of atmospheric turbulence. 

Recent work strongly relevant to this study includes studies that focus on using synthetic vision data to accurately locate the position of the runway and then detect moving objects on its surface [28], fusing real and virtual information to enhance airport scenes [32], and integrating EVS and SVS data to improve visibility in adverse atmospheric conditions [33]. All of these aim to enhance the visual information available to the pilot by some combination of real sensor information and virtual images produced by an SVS-like system, but none take the step of fusing the two directly. Thus, the work described in this paper differs from these, both because of the dual focus on runway and horizon detection, and because the direct fusion of EVS and SVS images is a novel approach to providing improved visual information of the runway scene to the pilot. The formulations of the steps to detect the runway and horizon, to fuse the EVS and SVS images, and to evaluate the performance subjectively (elements of which appeared in the PhD dissertation of Fadhil [34]) represent the principal contributions of this work.

## 2. EVS and SVS Image Registration

In general, image registration is the geometrical alignment of two or more images of the same scene. This can be particularly challenging when the images exist in different feature spaces [35]. Image registration algorithms can be classified into two groups: feature-based registration and area-based registration [36]. A feature-based registration method is employed in this study. As the goal of this study is to fuse the runways in each image as well as the surrounding areas, features for registration are derived from the runway corners and horizon end points. It is assumed that the runway is a quadrilateral composed of two long line segments and two shorter line segments and that the horizon is a long straight-line segment in both the EVS and SVS images. The runway corners can, therefore, be determined by detecting the end-points of the two long line segments and the horizon end-points can be determined from the line segment corresponding to the horizon. The first step, therefore, is to detect line segments in the EVS and SVS images and select those line segments that correspond to the two longer runway segments and the horizon line. As the SVS image quality is good and is unaffected by the weather conditions, the runway and horizon are first detected in the SVS image, and this information is used to estimate the runway and horizon in the corresponding EVS image. The $i^{\mathrm{th}}$ EVS and SVS frames are represented by $f_{E_{i}}\left(x,y\right)$ and $f_{S_{i}}\left(x,y\right)$, respectively, but because similar operations are performed on successive frames the formulations of the steps to register and fuse the SVS and EVS frames are simplified by dropping the subscript i.

### 2.1. SVS Runway and Horizon Detection

A careful examination of the SVS image frames reveals the following useful information: (i) the image is a simple gray-scale image, (ii) the runway is outlined by a bright boundary, and (iii) the horizon is a distinct boundary between the all-dark sky and the brighter non-sky regions. In the first step, the runway is detected by segmenting $f_{s}\left(x,y\right)$ according to:

(1) $$g_{s}\left(x,y\right)=\{\begin{array}{c} \begin{array}{cc} 1, & if\;f_{s}\left(x,y\right)>\left(\delta_{s}\right)Max\left[f_{s}\left(x,y\right)\right] \end{array}\\ \begin{array}{cc} 0, & \;Otherwise \end{array} \end{array}.$$

**Figure 2:**
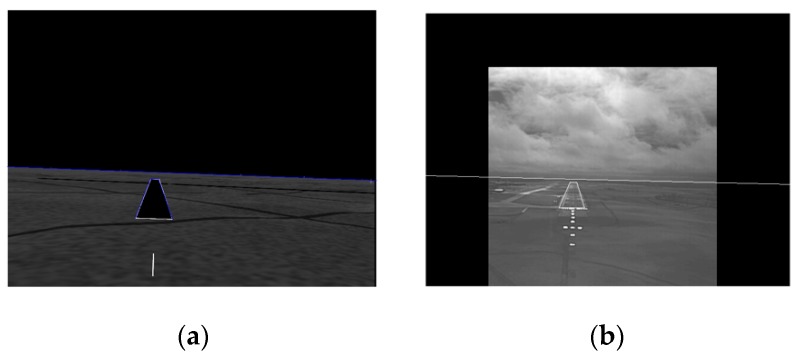
The runway and horizon detected in the (**a**) SVS image and (**b**) EVS image.

The factor $\delta_{s},0<\delta_{s}<1$ is determined empirically so that the resulting binary image $g_{s}\left(x,y\right)$ contains only the bright runway border. The four corner points of the runway are selected as runway registration control points in the SVS image. The end-points of the horizon, which will serve as horizon registration control points, are found by detecting the transition point from the dark pixels to the brighter pixels in the first and last columns of the image. The line joining the end-points defines the horizon in the SVS image. Figure 2a shows, in blue, the runway and horizon extracted from the SVS image in Figure 1 using $\delta_{s}=0.5$ which was found to give good segmentation results across all 1350 SVS frames. If $g_{s}\left(x,y\right)$ is the binary image of the runway and horizon, the angles of the two long runway line segments and the horizon line can be found from the co-linearity Hough transform {S(ρ,θ)} of $g_{s}\left(x,y\right)$. Let $\left\{S\left(\rho ,\theta_{1}\right)\right\}$ be the Hough transform accumulator cell with the highest count and let $\left\{S\left(\rho ,\theta_{2}\right)\right\}$, and $\left\{S\left(\rho ,\theta_{3}\right)\right\}$ be the cells with the next two highest counts. Then, the horizon angle is given by $\theta_{1}$ and the runway angles are given by $\theta_{2}$ and $\theta_{3}$. The angle parameters will be used to determine the runway lines and horizon in the EVS image. Additionally, the area $A_{s}$, of the runway quadrilateral is determined. As noted in the introduction, the focus is on registering and fusing the EVS and SVS images only when the aircraft is approaching the runway, so runway area is used as a parameter to indicate when the registration and fusion should begin. To make this parameter dimensionless, the parameter is normalized by dividing it by the frame area A, that is, $\left(A_{s}/A\right)$. Registration begins when the area ratio exceeds a threshold λ. This step may at first seem unnecessary, however there is clearly no need to register the images when the aircraft is far from the runway.

### 2.2. EVS Runway and Horizon Detection

Unlike the SVS image, the EVS image can be relatively complex, so the runway cannot be determined directly through segmentation and the horizon cannot be detected using the method developed for the SVS image. Moreover, the EVS frames are bound to be degraded with noise. In order to decrease the effects of noise, the EVS frames are filtered using a Weiner filter [37]. In the frequency domain, the EVS filtered image is given by

(2) $${\hat{F}}_{E}\left(u,v\right)=\left[\frac{1}{H\left(u,v\right)}\frac{{\left|H\left(u,v\right)\right|}^{2}}{{\left|H\left(u,v\right)\right|}^{2}+K}\right]F_{E}\left(u,v\right),$$

where H(u,v) is the degradation function $F_{E}\left(u,v\right)$, is the Fourier transform of $F_{E}\left(x,y\right)$, and K is a specified constant. The main degradation is assumed to be due to atmospheric turbulence, therefore, the function:

(3) $$H\left(u,v\right)=e^{-k{\left(u^{2}+v^{2}\right)}^{\frac{5}{6}}},$$

which is often used to model turbulence [37], is selected. The constant k can be adjusted according to the amount of turbulence.

The runway and horizon in the EVS image ${\hat{F}}_{E}\left(x,y\right)$ are detected using information extracted from the SVS image. The SVS runway and horizon serve as initial approximations for the EVS runway and horizon, respectively. In order to detect the runway in the EVS image, let ${\hat{r}}_{E}\left(x,y\right)$ be a rectangular region encompassing the approximated runway. The two long runway sides are within the diagonal and vertical orientations when the aircraft approaches the runway. Therefore, ${\hat{r}}_{E}\left(x,y\right)$ is converted into a region $r_{E}\left(x,y\right)$ containing vertical and approximately vertical lines by applying a (3 × 3) vertical line detection mask [37]. The region $r_{E}\left(x,y\right)$ is converted into a binary region $g_{E}\left(x,y\right)$ using the following segmentation rule:

(4) $$g_{E}\left(x,y\right)=\{\begin{array}{c} \begin{array}{cc} 1, & {\hat{r}}_{E}\left(x,y\right)>\left(\delta_{E}\right)Max\left[{\hat{r}}_{E}\left(x,y\right)\right] \end{array}\\ \begin{array}{cc} 0, & \;Otherwise \end{array} \end{array},$$

where, $\delta_{E}$ is determined empirically so that the resulting binary image contains only the bright line segments and removes the lower-intensity line segments. The Hough transform {E(ρ,θ)} of $g_{E}\left(x,y\right)$ is computed and the pixels contributing to accumulator cells $\left\{E\left(\rho ,\theta_{2}\pm \alpha \right)\right\}$, and $\left\{E\left(\rho ,\theta_{3}\pm \alpha \right)\right\}$, are selected to determine line segments that have approximately the same orientations as the SVS runway. The two largest line segments satisfying $\left\{E\left(\rho ,\theta_{2}\pm \alpha \right)\right\}$, and $\left\{E\left(\rho ,\theta_{3}\pm \alpha \right)\right\}$, are selected as the runway line segments in the EVS image. The parameter α is included to account for the fact that runway is not perfectly aligned in the SVS and EVS images. The end-points of these two line segments give the runway registration control points in the EVS image. Moreover, the lines connecting the runway control points define the estimated runway in the EVS image. The estimated runway, which tends to compactly enclose the actual runway, is superimposed onto the EVS image using an intensity equal to 255. In a similar manner, the horizon in the EVS image is estimated by using the SVS horizon as an initial approximation and finding the dominant line within $\left(\theta_{1}\pm \alpha_{1}\right)$ from the Hough transform in a band encompassing the initial approximation. The two-end points of the horizon give the horizon control points in the EVS image. The line joining the two end-points is superimposed on the EVs image using an intensity equal to 255. Figure 2b shows the estimated runway and horizon in the EVS image of Figure 1 using the following values for the parameters: k=0.0025, $\delta_{E}=0.6$, $\alpha =30^{o}$, $\alpha_{1}=25^{o}$. These values were found to give good results across the last 350 EVS frames that were involved in registration.

The horizon is assumed to be a straight line in the above formulations because of precedent in similar studies [29,30], and because the horizons in the data set used in this study are straight lines. The formulation can, however, be modified for the more general case in which the horizon is not a straight line. For example, the pixels forming the SVS horizon can be found by detecting the transition points from the dark pixels to the brighter pixels in all columns of the SVS image. This horizon then serves as the initial approximation in the EVS image, and the EVS horizon can then be determined in a band containing the initial approximation by detecting the sky-to-land transitions in each column of the EVS image.

### 2.3. Jitter Detection and Correction

The detection of the runway corners in the EVS image depends on its quality. Frame-to-frame changes can cause significant variation in the detected corner locations, resulting in ‘jitter’ in the detected runway across successive frames. This jitter is clearly undesirable because the detected runway should appear fixed while the aircraft is landing. In order to avoid this effect on the runway, the corners of the previous frame are used to determine if the corners in the current frame will lead to jitter. If $d_{i}$ is the distance between each pair of corner points $\left(x_{i},y_{i}\right)$ and $\left(x_{i-1},y_{i-1}\right)$ in the current and previous frames, respectively, and $A_{E}$ is the area of the runway in the EVS image, the normalized distance $\left(d_{i}/A_{E}\right)$ in the current and previous frame is compared with a threshold ρ. If $\left(d_{i}/A_{E}\right)$ exceeds the threshold, the corner point in the current frame is replaced with the corner point in the previous frame to prevent jitter. The median of the five previous corner points is used to update the previous frame corner points to ensure accuracy even when the corners in the previous frames are indistinct.

### 2.4. Horizon and Runway Registration

**Figure 3:**
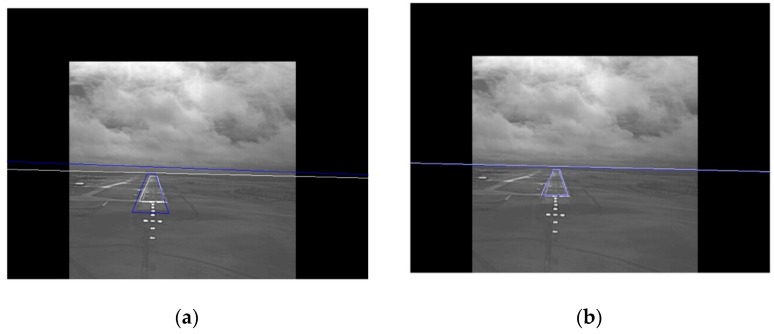
Superimposed SVS horizon and runway onto the EVS image with (**a**) no registration and (**b**) with registration.

Although not obvious visually, the runways and horizons in the SVS and EVS images are not aligned in Figure 2a,b. This is demonstrated in Figure 3a which shows the result of superimposing the SVS runway and horizon of Figure 2a onto Figure 2b. Clearly, the two images have to be registered prior to fusion. A two-step registration procedure was developed in which the two images are first globally aligned based on horizon registration before the runways are locally aligned within the horizon-aligned images. As the key information for landing is in the real EVS image, the SVS image is registered to the EVS image. That is, the SVS image is the target image and the EVS image is the reference image. The horizon corner points, top image corner points, and the bottom image corner points are the six pairs of control points selected for the horizon based registration. The runway corner points and the corner points of the runway encompassing rectangle r(x,y) are selected as the control points for registering the runways. The rectangle r(x,y) covers exactly the same regions in both images and is the also the same as region ${\hat{r}}_{E}\left(x,y\right)$ used to encompass the initial runway approximation in the EVS image. For both steps, the projective transformation is applied to register the images and the results of registering the images in Figure 2a,b are shown in Figure 3b. Note that the blue and white lines are perfectly aligned and almost appear as single blue lines. Additional registration examples will be presented in conjunction with the SVS and EVS fusion results.

### 2.5. Absence of Runway Approach-Line in the EVS and SVS Images

**Figure 4:**
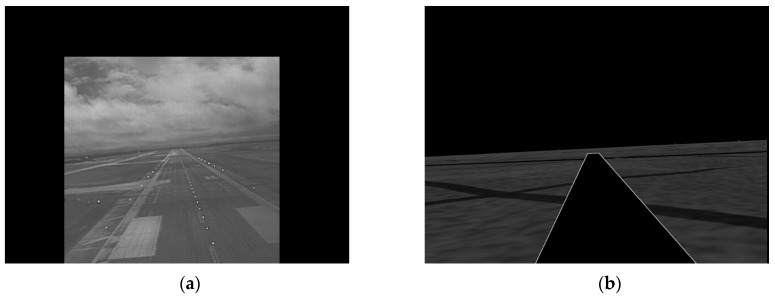
Frames which do not contain the runway approach-line: (**a**) EVS frame and (**b**) SVS frame.

Typically, the runway approach-line (the bottom line of the runway) is not visible in the SVS and EVS images when the aircraft is about to land on the runway as shown in Figure 4. This case is automatically detected when two corner points fall in the last row of the SVS image. At this stage of landing, the primary focus is on aligning and fusing the runways in the SVS and EVS images. The runway has a center line which pilots use to align the aircraft. This line is approximately vertical and can be determined from the Hough transform of the EVS image. A center line is generated in the SVS runway by connecting the midpoints of top and bottom corner points. The images are registered by aligning the pair of center lines. Examples will be shown in the experiments section.

## 3. EVS and SVS Image Fusion

Image fusion is the process of combining two or more images in such a way that information from both images is preserved [38,39]. The goal here is to generate such an image $W_{ES}\left(x,y\right)$ which is obtained by fusing the Weiner filtered EVS image $W_{E}\left(x,y\right)$ and the registered SVS image $W_{S}\left(x,y\right)$. The fused image is given by

(5) $$W_{ES}\left(x,y\right)=W_{E}\left(x,y\right)■W_{S}\left(x,y\right),$$

where ■ represents the fusion rule. Different aspects of the two images can be displayed in a single image by applying different fusion rules. The images can be fused directly in the spatial domain [39] or in a transform domain such as the wavelet domain [38]. Although quite simple, the spatial domain rules are limited to the global application of operations such as pixel averaging or maximum selection [40,41]. Conversely, the wavelet domain rules offer greater flexibility in developing fusion rules. For example, different fusion rules can be applied to combine the wavelet sub-bands [39,40,41,42,43,44]. This flexibility is the primary reason for selecting and developing fusion rules based on the DWT in this study. In most fusion applications, equal weighting is given to both images in the sense that the fused image should capture the information from both images equally. Consequently, the correlation between the fused image and the two original images is often used as a measure to evaluate the fusion performance [41,42,45]. Feature ranking or weighting based on correlation accuracy or mechanistic assessment, however, can significantly enhance fusion classification [46]. In this study equal weighting is not assumed because the EVS image is more important than the “supplementary” information in the SVS image. If the dimension of $W_{E}\left(x,y\right)$ is assumed to be M × N, the DWT of $W_{E}\left(x,y\right)$ can be written as

(6) $$W_{E}^{a}\left(m,n\right)=\frac{1}{\sqrt{MN}}\overset{M-1}{\underset{x=0}{\sum }}\overset{N-1}{\underset{y=0}{\sum }}w_{E}\left(x,y\right)\varphi_{m,n}\left(x,y\right),$$

(7) $$W_{E}^{i}\left(m,n\right)=\frac{1}{\sqrt{MN}}\overset{M-1}{\underset{x=0}{\sum }}\overset{N-1}{\underset{y=0}{\sum }}w_{E}\left(x,y\right)\psi_{m,n}^{i}\left(x,y\right),i=\left\{h,v,d\right\},$$

where the scaled and translated basis functions are given by

(8) $$\varphi_{m,n}\left(x,y\right)=2^{j/2}\left(x,y\right)\varphi \left(2^{j}x-m,2^{j}y-n\right),$$

(9) $$\psi_{m,n}^{i}\left(x,y\right)=2^{j/2}\psi^{i}\left(2^{j}x-m,2^{j}y-n\right),i=\left\{h,v,d\right\}.$$

The basis functions are assumed to be separable and can, therefore, be written as

(10) $$\varphi_{m,n}\left(x,y\right)=\varphi \left(x\right)\varphi \left(y\right),$$

(11) $$\psi_{m,n}^{i}\left(x,y\right)=\psi \left(x\right)\psi \left(y\right),i=\left\{h,v,d\right\}.$$

Extensive experiments in a notable study by Zheng et al. [43] have shown that different basis functions tend to give similar fusion results. The Haar wavelet transform is selected in this study because of its computational simplicity. For the Haar transform, the one-dimensional scaling and wavelet vectors are given by

(12) $$\varphi \left(x\right)=\{\begin{array}{c} \begin{array}{cc} \frac{1}{\sqrt{2}}, & x=0,\;1 \end{array}\\ \begin{array}{cc} 0, & \mathrm{Otherwise} \end{array} \end{array},$$

(13) $$\psi \left(x\right)=\{\begin{array}{c} \begin{array}{cc} \frac{1}{\sqrt{2}}, & x=0 \end{array}\\ \begin{array}{cc} \frac{-1}{\sqrt{2}}, & x=1 \end{array}\\ \begin{array}{cc} 0, & \mathrm{Otherwise} \end{array} \end{array}.$$

The corresponding inverse wavelet transform is then given by

(14) $$w_{E}\left(x,y\right)=\frac{1}{\sqrt{MN}}\left[\overset{\frac{M}{2}-1}{\underset{m=0}{\sum }}\overset{\frac{N}{2}-1}{\underset{n=0}{\sum }}W_{E}^{a}\left(m,n\right)\varphi_{m,n}\left(x,y\right)+\underset{i=h,v,d}{\sum }\overset{\frac{M}{2}-1}{\underset{m=0}{\sum }}\overset{\frac{n}{2}-1}{\underset{n=0}{\sum }}W_{E}^{i}\left(m,n\right)\psi_{m,n}^{i}\left(x,y\right)\right],$$

where $W_{E}^{a}\left(m,n\right)$, $W_{E}^{v}\left(m,n\right)$, $W_{E}^{h}\left(m,n\right)$, and $W_{E}^{d}\left(m,n\right)$ are the four (M/2) × (N/2) sub-bands of the DWT of $w_{E}\left(x,y\right)$. These sub-bands are the approximation, vertical detail, horizontal detail, and diagonal detail sub-bands of $w_{E}\left(x,y\right).$ Similarly $W_{S}^{a}\left(m,n\right)$, $W_{S}^{v}\left(m,n\right)$, $W_{S}^{h}\left(m,n\right)$, and $W_{S}^{d}\left(m,n\right)$ represent the sub-bands of the DWT of $w_{S}\left(x,y\right)$. Using the DWT sub-bands the images can be fused according to the following rules:

Maximum Selection Rule

(15) $$W_{ES}^{a}\left(m,n\right)=MAX\left[W_{E}^{a}\left(m,n\right),W_{S}^{a}\left(m,n\right)\right],\;W_{ES}^{v}\left(m,n\right)=MAX\left[W_{E}^{v}\left(m,n\right),W_{S}^{v}\left(m,n\right)\right],\;W_{ES}^{h}\left(m,n\right)=MAX\left[W_{E}^{h}\left(m,n\right),W_{S}^{h}\left(m,n\right)\right],\;W_{ES}^{d}\left(m,n\right)=MAX\left[W_{E}^{d}\left(m,n\right),W_{S}^{d}\left(m,n\right)\right].$$

Average Selection Rule

(16) $$W_{ES}^{a}\left(m,n\right)=AVG\left[W_{E}^{a}\left(m,n\right),W_{S}^{a}\left(m,n\right)\right],\;W_{ES}^{v}\left(m,n\right)=AVG\left[W_{E}^{v}\left(m,n\right),W_{S}^{v}\left(m,n\right)\right],\;W_{ES}^{h}\left(m,n\right)=AVG\left[W_{E}^{h}\left(m,n\right),W_{S}^{h}\left(m,n\right)\right],\;W_{ES}^{d}\left(m,n\right)=AVG\left[W_{E}^{d}\left(m,n\right),W_{S}^{d}\left(m,n\right)\right].$$

Mixed Selection Rule

(17) $$W_{ES}^{a}\left(m,n\right)=AVG\left[W_{E}^{a}\left(m,n\right),W_{S}^{a}\left(m,n\right)\right],\;W_{ES}^{v}\left(m,n\right)=MAX\left[W_{E}^{v}\left(m,n\right),W_{S}^{v}\left(m,n\right)\right],\;W_{ES}^{h}\left(m,n\right)=MAX\left[W_{E}^{h}\left(m,n\right),W_{S}^{h}\left(m,n\right)\right],\;W_{ES}^{d}\left(m,n\right)=MAX\left[W_{E}^{d}\left(m,n\right),W_{S}^{d}\left(m,n\right)\right].$$

Modified Selection Rule

(18) $$W_{ES}^{a}\left(m,n\right)=W_{ES}^{a}\left(m,n\right),\;W_{ES}^{v}\left(m,n\right)=MAX\left[W_{E}^{v}\left(m,n\right),W_{S}^{v}\left(m,n\right)\right],\;W_{ES}^{h}\left(m,n\right)=MAX\left[W_{E}^{h}\left(m,n\right),W_{S}^{h}\left(m,n\right)\right],\;W_{ES}^{d}\left(m,n\right)=MAX\left[W_{E}^{d}\left(m,n\right),W_{S}^{d}\left(m,n\right)\right],$$

where $W_{ES}^{a}\left(m,n\right)$, $W_{ES}^{v}\left(m,n\right)$, $W_{ES}^{h}\left(m,n\right)$, and $W_{ES}^{d}\left(m,n\right)$ are the sub-bands of the EVS-SVS fused image. The fused image $W_{ES}\left(x,y\right)$ is obtained from the inverse wavelet transform after application of the selection rule. Note that most often, images are fused using the maximum, average, or mixed rules [39,40,41,42,43,44]. The modified rule is introduced to give more weight to the EVS image by preserving the EVS information in the approximation band and can be regarded as a modification of the maximum and mixed rules. All four fusion rules are implemented and evaluated in the following section.

## 4. Registration and Fusion Experimental Results

This section describes the experiments designed to evaluate the overall procedure of registering and the EVS and SVS image frames. The data set, provided by Rockwell-Collins, consists of 1350 EVS and SVS image pairs acquired from the infra-red sensor on an aircraft and a satellite, respectively. The dimensions of EVS and SVS frames are 1050 × 1400 and the frame rate was 6 fps. The following values were used for the parameters: $\delta_{s}=0.5$, λ=0.00885, k=0.0025, $K=1\times 10^{-8}$, $\delta_{E}=0.6$, $\alpha =30^{o}$, $\alpha_{1}=25^{o}$, $\rho =1\times 10^{-5}$. After setting the parameters, the entire sequence of frame pairs was processed autonomously without any intervention.

**Figure 5:**
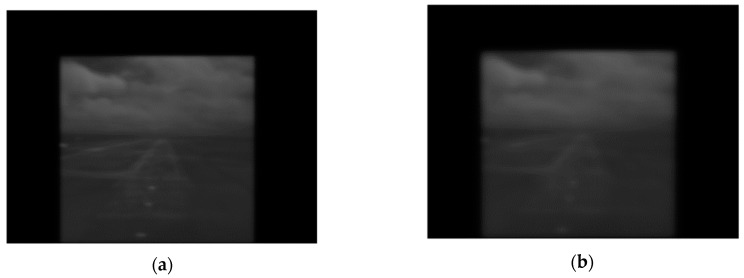
(**a**) EVS image with intermediate turbulence (*k* = 0.001) and (**b**) EVS images with severe turbulence (*k* = 0.0025).

Clearly, it is not practical to show the results for all 1350 frame pairs, therefore, only a few representative examples which show different stages of approach and landing are shown to conduct subjective evaluations of runway visibility. Additionally, the real data set does not cover varying weather conditions, so in order to conduct more detailed subjective and objective evaluations, data sets containing various levels of atmospheric turbulence in the EVS frames were generated using the same degradation model H(u,v) used to filter the EVS image. Two sets of EVS frames were generated to simulate intermediate level turbulence using k=0.001, and severe level turbulence using k=0.0025 in each frame. Examples of degraded EVS frames are shown in Figure 5. The registration performance can thus be evaluated numerically by the root-mean-square (rms) error between the manually detected runway corner points and horizon points in the EVS image, selected using mouse-clicks, and the corner points detected by the registration procedure.

**Figure 6:**
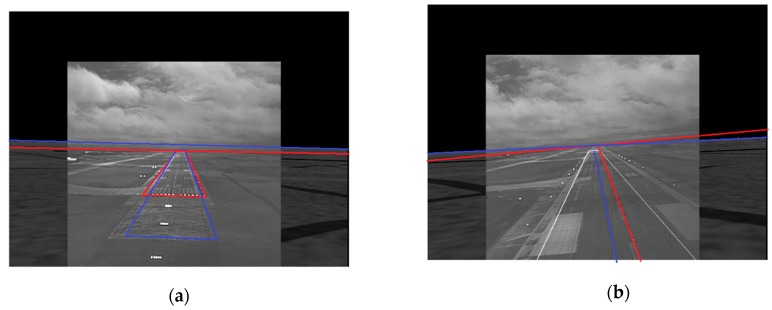
Examples EVS-SVS image fusion without prior registration. (**a**) EVS and SVS runways and horizons are not aligned; (**b**) EVS and SVS horizons and center lines are not aligned.

**Figure 7:**
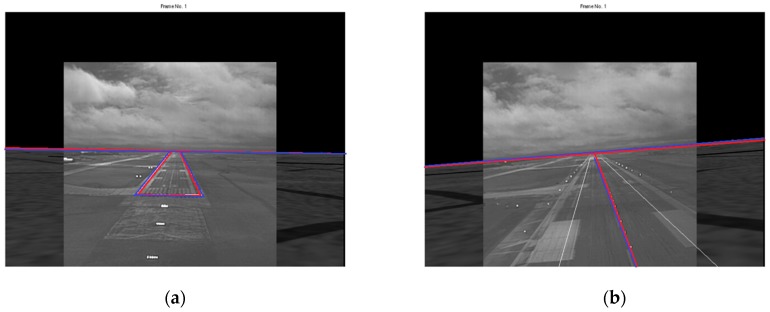
Examples of EVS-SVS image fusion with registration. (**a**) EVS and SVS runways and horizons are aligned; (**b**) EVS and SVS horizons and center lines are aligned.

Figure 6 shows examples of fusing the EVS and SVS images directly using the maximum fusion rule without prior registration. The runways, horizons, and center lines in the SVS and EVS images are shown in blue and red, respectively, to facilitate visual analysis. Observe the following problems: (i) the runways and horizons are not aligned in Figure 6a, (ii) the horizons and runway center lines are not aligned in Figure 6b, and (iii) the SVS runway is much larger than the EVS runway in Figure 6a. Similar problems are observed with the four other fusion rules. Figure 7 shows the fusion results of the same images after they were registered. Observe that the runways, center lines, and horizons are aligned quite well.

## 5. Comparison of Fusion Methods

**Figure 8:**
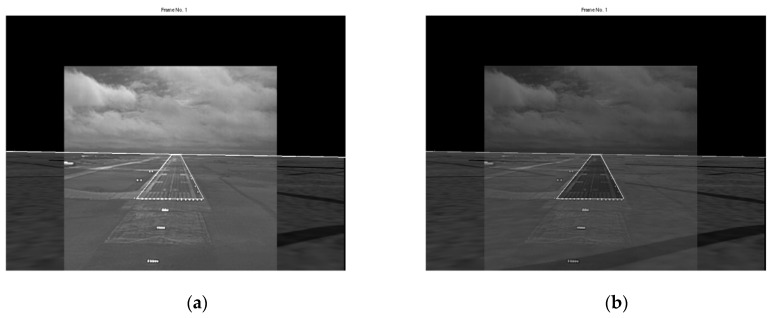

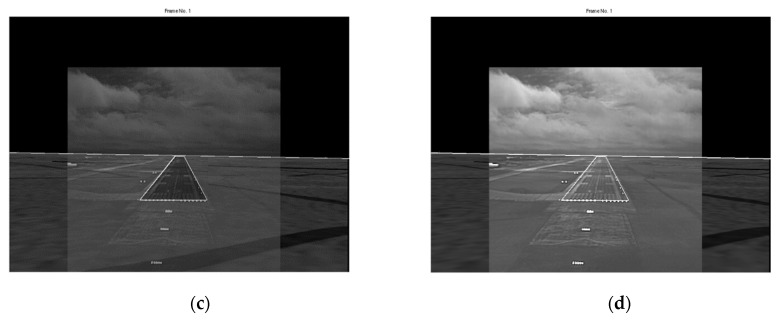
Fusion in no-turbulence (*k* = 0). (**a**) Maximum rule; (**b**) Average rule; (**c**) Mixed rule; and (**d**) Modified rule.

**Figure 9:**
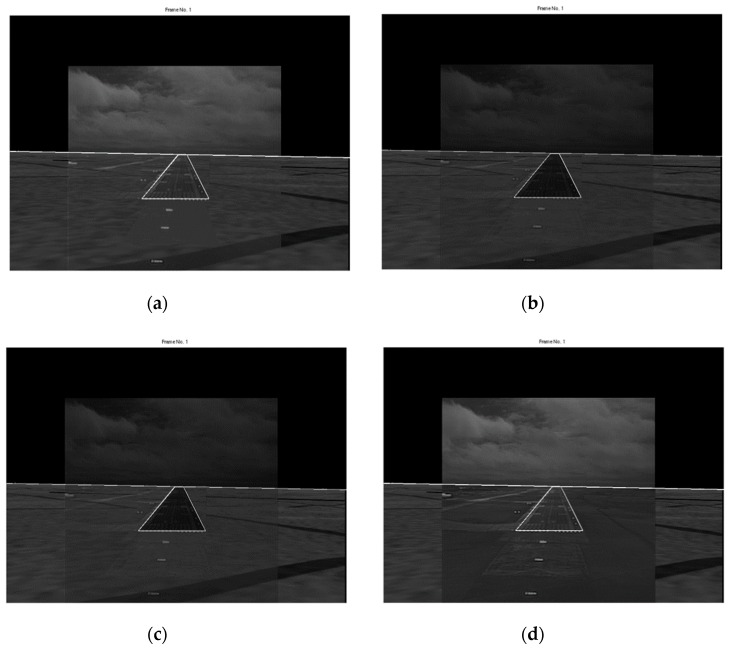
Fusion in severe turbulence (*k* = 0.0025). (**a**) Maximum rule; (**b**) Average rule; (**c**) Mixed rule; and (**d**) Modified rule.

Examples of fusing pairs of registered EVS and SVS images using the four fusion rules are shown in Figure 8 and Figure 9 for the original (no-turbulence assumption) and severe turbulence cases, respectively. The most important results are for the severe turbulence case because there is not much need for runway and horizon detection when there is little or no atmospheric turbulence. The no-turbulence results are included to draw general conclusions relating to the characteristics of the images combined by using different fusion rules. It is interesting to observe the following characteristics in the results:

(a) The results for the no-turbulence case show subtle but expected differences between the four rules. The maximum selection rule tends to favor the brighter image in the approximation band which is typically the EVS image. Selecting the maximum in the other sub-bands tends to sharpen the edges in the fused image. Consequently, as evident in Figure 8a, the runway and horizon appear brighter in the fused image. The average selection rule preserves details from both images with equal weighting. However, there is a slight loss in contrast throughout the fused image due to smoothing which is typical of averaging. Moreover, averaging the approximation bands, which are low frequency bands, will tend to blur the low frequencies which is undesirable. A careful examination of Figure 8b confirms this loss in contrast in the entire image. The mixed selection rule preserves the EVS and SVS approximation details with equal weighting while sharpening the high frequencies. As a result, the fused image in Figure 8c appears to be sharper, however, there is a slight loss in the contrast due to averaging the approximation sub-bands. The modified selection rule preserves the EVS approximation information completely while enhancing the high frequency information. Consequently, the fused image in Figure 8d is sharper and looks similar to the maximum rule which favors the brighter EVS image.
(b) The general characteristics observed for the no-turbulence case also hold for the severe turbulence case, however, there are some important differences across the four fusion results shown in Figure 9. For example, the maximum rule tends to preserve more of the SVS information, as seen in Figure 9a because the SVS image is brighter than the EVS image. This is quite undesirable. The results for the average and mixed rules shown in Figure 9b,c, respectively, are also quite unsatisfactory because of the overall decrease in contrast. The best result, shown in Figure 9d, is obtained using the modified rule because it preserves the important EVS information while also fusing the lesser important SVS information. Most importantly, the runway details are the clearest in Figure 9d which can also be useful for detecting obstacles on runways.

**Figure 10:**
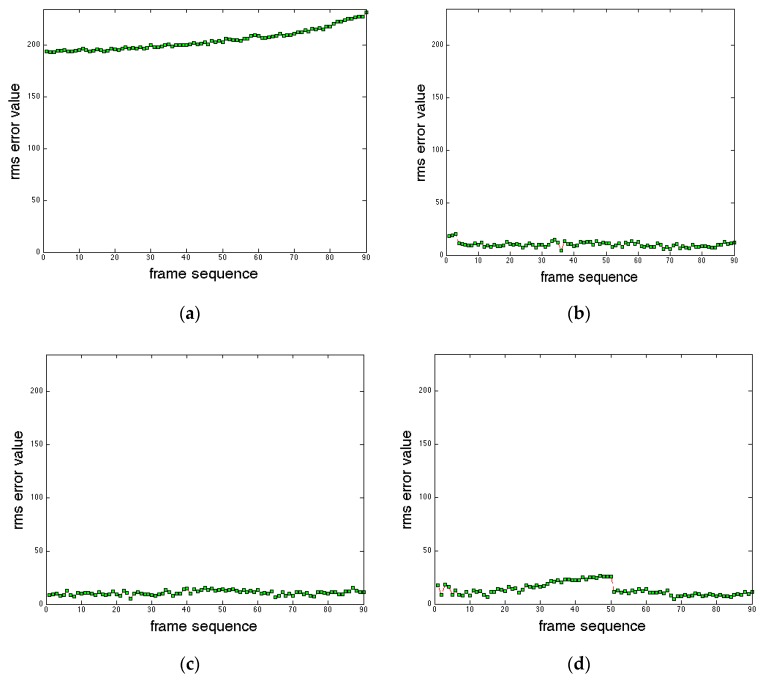
Corner and horizon registration rms errors. (**a**) Original data set with no registration; (**b**) original data set with registration; (**c**) data set with intermediate turbulence; and (**d**) data set with severe turbulence.

Although the differences in the fused images are subtle, the observed characteristics can be used as a guide for selecting fusion rules based on what aspects of the two images need to be emphasized in various applications. The next set of results are included to evaluate the registration performance by evaluating the rms distance error between the locations of the manually detected runway and horizon corners points and locations of the registered runway and horizon corner points. Figure 10 summarizes the rms errors obtained from the original data set without registration, the original data set with registration, and the two simulated degraded data sets with registration. The results cover frames 1101 to 1190. The frames are ordered such that the frame number increases as the distance between the aircraft and runway decreases. The original data set without registration is included to compare the registration performance.

The following points should be noted regarding the results presented in Figure 10, which are representative of the registered results overall:

(a) When no registration is employed, the rms error is relatively high. Moreover, the rms error increases when the distance between the aircraft and the runway decreases. This increase is clearly undesirable because it is even more critical to detect the runway accurately as the aircraft gets closer to the runway.
(b) Employing registration noticeably reduces the rms error overall but, most importantly, the errors do not increase when the aircraft gets closer to the runway. Furthermore, the increase in the rms error in the presence of severe turbulence is marginal and only lasts for the first 50 s before dropping to similar levels with the intermediate turbulence case.
(c) The error trends across the registered results are quite similar. Furthermore, the increase in the rms errors in the presence of severe turbulence is marginal.
(d) No parameters were changed between the four sets of results, and no attempt was made to adjust the parameters to accommodate the different levels of turbulence in Figure 10c,d.
(e) Although it is not possible to show in still images, the jitter is observed to be minimal when the registered and fused frames are displayed as a video at the frame capture rate, corresponding to a pilot’s subjective experience of the fused data stream.

### Real Time Implementation

In order to establish the veracity of implementing the algorithm for real time applications, the time needed for each frame to be processed was calculated. The algorithm was written in Matlab code in such a way to minimize execution time, and run on an HP Laptop with Intel Processor, core i7, 2.7 GHz and 8 GB Ram. The average processing time for three representative frame sequences is shown in Table 1. 

These times are sufficient in their current form and comparable to the fastest fusion algorithm trailed by Kumar et al. [33], which had an execution time of 0.4158 s. While our algorithm does not match that benchmark under the aforementioned testing conditions, run times given are in Matlab which is an interpreted language. Real-time use would be executed in a compiled language with dedicated hardware, which would significantly increase execution speed and easily meet requirements for real-time use.

## 6. Discussion

Image fusion between EVS and SVS represents an underexplored field of investigation today, perhaps because of an intuition that real and virtual images should be kept separate; directly comparable studies are very difficult to find. A few investigations have attempted to enhance the visual information available to aircraft pilots using both real and virtual images, but the virtual images are either used as a reference to assist visual processing performed on the real images [28] or as a benchmark for fused visual spectrum/infrared images [32,33]. Nonetheless, due to the shared goals of these studies, some functional comparisons can be made. Hamza et al. [28] used synthetic data to provide a predicted location for the runway before detecting it in the EVS image, but this is only a necessary prior step to creating a model of the runway that can be used to detect occluding moving objects. As such, only the first step of the procedure resembles the system described in this study. The fused images demonstrated by Cheng et al. in [32] combine the ability of visual cameras to capture runway lights with infrared cameras’ ability to clearly image the surrounding area in low light conditions. The method presented here dispenses with the need to image runway lights by obtaining the high-contrast outline of the runway from the SVS images. Under optimal weather conditions this is effectively equivalent, as both systems provide a high level of detail for the both the runway and its surroundings. The weather-independent nature of the SVS, however ensures that the runway detection will not degrade even in high turbulence (Figure 9). Similar considerations apply to the work of Kumar et al. [33]. Although data used is taken from a ground vehicle, the goals of simultaneously imaging runway lights and unlit features such as markings on the runway tarmac are essentially the same. In both these studies a GPS-linked virtual image is used to validate the results and as an alternative information stream, but it does not form part of the fused image.

As the fused EVS/SVS images produced in this study are intended to assist a pilot or drone operator in landing an aircraft, the system can only be judged subjectively. This evaluation takes the form of demonstrating that the runway remains highly visible in the fused images despite turbulence, but ultimately it must be demonstrated that the user’s ability to land an aircraft is actually enhanced when viewing the fused images. While testing such a system on a real aircraft in actual turbulent conditions entails obvious risks, in the future it could be tested by incorporating the system into a flight simulator and evaluating user performance against standard training metrics.

## 7. Conclusions

An innovative procedure was introduced to accurately detect runways and horizons as well as enhance surrounding runway areas by fusing EVS and SVS images. Central to the procedure is a mathematical architecture for fusion of EVS and SVS runway images captured from an aircraft in the final stages of landing. A registration algorithm was further introduced to align the EVS and SVS images prior to fusion. The most notable feature of the registration is its guidance is based solely on information extracted from weather-invariant SVS images enabling utility in areas where enhanced information is not available. Four fusion rules based on combining DWT sub-bands were implemented, contrasted, and evaluated. The resulting procedure was tested on real EVS-SVS image pairs and also on image pairs containing simulated EVS images with varying levels of turbulence. Subjective and objective evaluations reveal that runways can be detected accurately, even in poor visibility conditions due to severe levels of atmospheric turbulence. It was also demonstrated that different aspects of the EVS and SVS images can be emphasized by use of different DWT-based fusion procedures. The modified rule was judged to yield the best fusion results for the problem under consideration. Another notable feature is that the entire procedure is autonomous throughout the landing sequence, irrespective of the weather conditions; the fixed parameters are set initially, and variable parameters are determined automatically from image frames during operation. Given the excellent fusion results and autonomous functionality, we conclude the fusion procedure developed holds strong potential for incorporation into head-up displays (HUDs) to assist pilots in safely landing aircraft in varying weather conditions. It can also be implemented to help remotely piloted UAVs land in challenging conditions in remote locations. Furthermore, the procedure developed can be easily modified to fuse image pairs in other visual processing applications. Translation is also envisioned where the system works in conjunction with other novel auto piloting systems [47]. The results can also serve as a guide for selecting different signal fusion rules for many other signal fusion applications. 

## Figures and Tables

**Table 1:** Average execution time of registration and fusion algorithm over three representative frame sequences.

Frame Sequence	Average Processed Time (s)
1100:1180	1.0938
1181:1225	1.0850
1226:1349	1.1446
